# Isolation and characterisation of *Leishmania donovani* protein antigens from urine of visceral leishmaniasis patients

**DOI:** 10.1371/journal.pone.0238840

**Published:** 2020-09-14

**Authors:** Tegwen Marlais, Tapan Bhattacharyya, Callum Pearson, Bathsheba L. Gardner, Safiyyah Marhoon, Stephanie Airs, Kiera Hayes, Andrew K. Falconar, Om Prakash Singh, Steven G. Reed, Sayda El-Safi, Shyam Sundar, Michael A. Miles

**Affiliations:** 1 Department of Clinical Research, Faculty of Infectious and Tropical Diseases, London School of Hygiene and Tropical Medicine, London, United Kingdom; 2 Department of Infection Biology, Faculty of Infectious and Tropical Diseases, London School of Hygiene and Tropical Medicine, London, United Kingdom; 3 Departamento de Medicina, Universidad del Norte, Barranquilla, Colombia; 4 Department of Biochemistry, Institute of Science, Banaras Hindu University, Varanasi, Uttar Pradesh, India; 5 Infectious Disease Research Institute, Seattle, Washington, United States of America; 6 Faculty of Medicine, University of Khartoum, Khartoum, Sudan; 7 Department of Medicine, Institute of Medical Sciences, Banaras Hindu University, Varanasi, Uttar Pradesh, India; Universidade Federal da Bahia, BRAZIL

## Abstract

Diagnosis of visceral leishmaniasis (VL) relies on invasive and risky aspirate procedures, and confirmation of cure after treatment is unreliable. Detection of *Leishmania donovani* antigens in urine has the potential to provide both a non-invasive diagnostic and a test of cure. We searched for *L*. *donovani* antigens in urine of VL patients from India and Sudan to contribute to the development of urine antigen capture immunoassays. VL urine samples were incubated with immobilised anti-*L*. *donovani* polyclonal antibodies and captured material was eluted. Sudanese eluted material and concentrated VL urine were analysed by western blot. Immunocaptured and immunoreactive material from Indian and Sudanese urine was submitted to mass spectrometry for protein identification. We identified six *L*. *donovani* proteins from VL urine. Named proteins were 40S ribosomal protein S9, kinases, and others were hypothetical. Thirty-three epitope regions were predicted with high specificity in the 6 proteins. Of these, 20 were highly specific to *Leishmania* spp. and are highly suitable for raising antibodies for the subsequent development of an antigen capture assay. We present all the identified proteins and analysed epitope regions in full so that they may contribute to the development of non-invasive immunoassays for this deadly disease.

## Introduction

Visceral leishmaniasis (VL) is most commonly caused by *Leishmania donovani* in the Indian subcontinent and eastern Africa, whereas *L*. *infantum* is the agent in the Mediterranean, Middle East and South America. Both species are transmitted by female phlebotomine sand flies and symptomatic infection is considered fatal if untreated, therefore accurate diagnosis is crucial to patient outcome. India, Bangladesh and Nepal are aiming to eliminate VL as a public health problem and this relies on rapid case detection and confirmation of cure after treatment [1].

Routine diagnosis of VL is based on serology, commonly using the recombinant rK39 or rK28 antigens, followed by microscopic visualisation of the parasite in spleen, bone marrow or lymph node aspirate as confirmation. Conventional serology, which detects anti-*Leishmania* IgG, has several drawbacks: it is ineffective at confirming cure or relapse because it can remain positive for many years after successful treatment [2–6]; it is also less reliable in HIV co-infected cases where a negative result does not rule out leishmaniasis [7]. Molecular assays are sometimes applied, and may gain increased importance during VL elimination, however, non-invasive antigen detection would complement this as a diagnostic tool [8].

An ideal diagnostic for both primary VL cases and validating cure is the detection of parasite material in non-invasive samples such as urine or saliva, or a serological test that is specific for active infection [9]. As well, there is the need for low-cost, rapid and equipment-free diagnostics that can be used in low-resource settings at point-of-care with minimal training. Such assays may detect parasite DNA, for example by loop-mediated isothermal amplification (LAMP) [10, 11] or by recombinase polymerase amplification (RPA) [12], or may detect parasite antigens.

Several urine antigen capture immunoassays have been developed with the best established being the KAtex, a latex particle agglutination test that detects a carbohydrate antigen [13, 14]. The KAtex has a specificity of 84–100%, but poorer sensitivity of 47–87% [15–18] with the drawback that urine samples must be boiled before testing. However, the test is rapid, giving a result in less than 10 minutes and becoming negative for most patients 30 days post-treatment [18]. In addition, this urine antigen assay has shown utility in HIV/VL co-infection [19, 20]. Monoclonal and polyclonal antibodies against the undefined antigen in the KAtex test were later adapted to ELISA format [21].

Other assays have been reported that detect particular protein antigens of *L*. *infantum* in urine [22]. This approach required first identifying *Leishmania* proteins in VL urine by mass spectrometry, expressing them as recombinant antigens and raising antibodies that could be produced as highly specific and sensitive polyclonal or monoclonal antibodies [22].

An alternative approach is to raise antibodies to lysed whole parasite cells, containing a wide diversity of antigens and to use these to capture a range of undefined antigens from VL patient urine. Vallur et al. [23] reported the development of an ELISA using an affinity purified polyclonal rabbit antibody against *L*. *donovani* whole cell lysate. The assay was optimised by those authors and developed into an ELISA kit that performed well in detecting urine antigen in VL patients from both *L*. *infantum* and *L*. *donovani* endemic regions. Here we have undertaken a study using this antibody and other polyclonal anti-*Leishmania* antibodies, and by mass spectrometry, we have identified antigens in Indian and Sudanese VL urine, for the development of antigen capture assays.

## Materials and methods

### Ethics statement

Ethical permission was granted by the LSHTM Ethical Review Committee with approval number 11478, and as part of the EC-funded NIDIAG project. In India, the collection of samples was approved by the Ethics Committee of Banaras Hindu University, Varanasi. In Sudan, approval was by the Ethical Research Committee, Faculty of Medicine, University of Khartoum and the National Health Research Ethics Committee, Federal Ministry of Health, Sudan. Written informed consent was obtained from adult subjects included in the study or from the parents or guardians of individuals less than 18 years of age.

Two rabbits were used here to raise antiserum. Rabbit sera were produced in compliance with UK Home Office regulations and the Animals (Scientific Procedures) Act 1986, in authorised animal facilities, by licensed staff, and in accord with European regulations and the 3R policy of Refinement, Reduction and Replacement.

### Processing and analysis of samples

**Fig 1:**
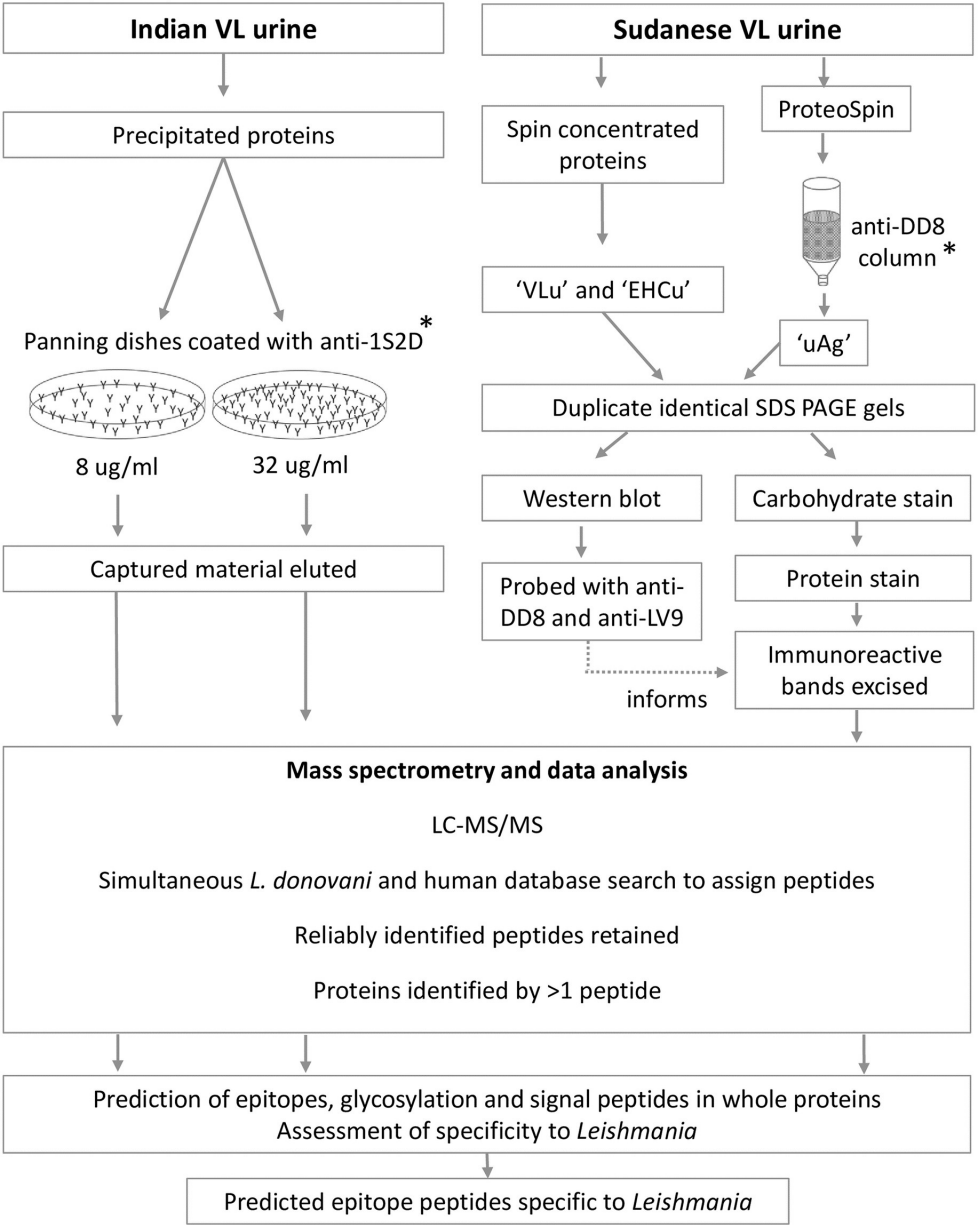
Sample processing and analysis workflow used in this study. Asterisks indicate immunocapture methods. Anti-1S2D, -DD8 and -LV9 are rabbit antibodies against these respective strains of *L*. *donovani*. VLu, concentrated proteins from VL urine; EHCu, concentrated proteins from urine of Endemic Healthy Controls; uAg, material captured from VL urine by anti-*L*. *donovani* antibodies; LC-MS/MS, liquid chromatography tandem mass spectrometry.

Fig 1 depicts the overall workflow of the urine samples in this study. We used two distinct methods in order to maximise the possibility of antigen capture.

### Urine samples

Urine samples were from VL patients and healthy controls in two distinct VL endemic regions, namely the Indian state of Bihar and the Sudanese state of Gedaref. India: urine samples were collected from VL patients attending the Kala-Azar Medical Research Centre (KAMRC) clinic in Muzaffarpur, Bihar. Sudan: VL and endemic healthy control (EHC) urine samples were collected from field sites in Gedaref. In this work *Leishmania*-derived antigens were enriched from VL urine using antibodies, prior to mass spectrometry.

### Generation of rabbit anti-*L*. *donovani* antibodies

Vallur et al. [23] described the generation of a rabbit anti-*L*. *donovani* polyclonal antibody against whole promastigote lysate antigen, which was affinity purified against soluble promastigote lysate antigen of *L*. *donovani* strain MHOM/SD/00/1S-2D. This antibody is hereafter referred to as anti-1S2D.

Here, we prepared soluble promastigote lysate antigens of *L*. *donovani* strains MHOM/IN/80/DD8 and MHOM/ET/67/LV9 (also known as HU3), as described [24]. Log phase promastigotes were washed 3 times in PBS, pelleted by centrifugation, flash frozen in liquid nitrogen and thawed 3 times, sonicated (Soniprep 150, MSE, UK) at 12 microns intensity for 3 x 30 seconds at 2-minute intervals on ice and finally centrifuged at 12,000 *x g* for 1 minute at 4°C. The supernatants containing soluble antigens were retained and used subsequently.

Soluble antigen (DD8 and LV9) was used to raise antiserum in two rabbits that were immunised percutaneously with two doses of the respective lysate. The first inoculation was with 200 μg—500 μg of lysate antigen (originating from approximately 10^7^ parasites) with Freund’s complete adjuvant (F5881, Sigma-Aldrich). A second inoculation, four weeks later, was with the same amount of antigen, plus Freund’s incomplete adjuvant (F5506, Sigma-Aldrich). Five millilitres of blood was taken before inoculations and about 50 ml five months after the second inoculation.

The resulting polyclonal IgG was purified from both DD8 and LV9 antisera on protein A agarose (P3476, Sigma, UK), eluted with 0.1 M glycine, pH 2.7 and immediately neutralised with 60 μl of 1 M Tris pH 9.0. The purified IgG elutions are hereafter referred to as anti-DD8 and anti-LV9. An ELISA was performed to confirm the reactivity of the two antibodies with *L*. *donovani* antigen and that the pre-immune sera were non-reactive.

### Indian VL urine

#### Precipitation of urine proteins

Total protein was precipitated from Indian VL patient urine: approximately 130 ml of urine (83 ml fresh and 52 ml previously frozen) was combined from 31 Indian VL patients and protease inhibitor cocktail (P8340, Sigma-Aldrich) was added at a dilution of 1:500. The urine was centrifuged at 2,500 x g for 3 minutes to pellet cellular debris, which was discarded. To the supernatant, we added 85 g solid ammonium sulphate to achieve 90% saturation then incubated the solution for one hour at ambient temperature (about 28°C) with mixing to allow proteins to precipitate. The urine/ammonium sulphate solution was centrifuged in aliquots at 3,900 *x g* at 20°C for 40 minutes and the supernatant was discarded. Protein pellets were then resuspended as two aliquots in a total of 4.25 ml PBS then desalted using PD10 columns (170851–01, GE Healthcare) by the centrifugation protocol and each replicate made up to 3 ml with PBS.

#### Immunocapture of proteins

To capture *L*. *donovani* antigens from the precipitated Indian VL urine protein, we performed immuno-panning (Fig 1, upper left). Anti-1S2D antibody was coated onto 5 cm diameter plastic dishes at 8 μg/ml (Dish 8) and, to increase antigen capture, at 35 μg/ml (Dish 35) in 1.5 ml coating buffer (0.1 M NaHCO_3_, pH 8.6) by incubation at 4°C overnight. The unbound antibody was removed, replaced with blocking buffer (coating buffer containing 5 mg/ml bovine serum albumin) and the dishes were incubated at 4°C for 1 hour. After blocking, both dishes were washed 5 times using PBS containing 0.05% v/v Tween 20 (PBST), with gentle agitation.

The first aliquot of urine protein solution was incubated in Dish 8 for 1 hour at room temperature with agitation before the unbound material was moved into Dish 35. Dish 8 was washed 3 times with PBST then another 3 times with PBS before bound material was eluted with a 15 minute incubation with 1 ml of 0.1 M glycine pH 2.7. Eluate was pipetted into a tube containing 32 μl neutralising buffer (1 M Tris pH 9.0) and sodium azide to a final concentration of 0.02% (w/v) then stored at -80°C.

Unbound material from Dish 35 was transferred back to Dish 8 for repetition of the whole process. From each of the two aliquots of urine protein solution, two elutions were made from each of the two dishes (thus n = 8 eluates), to maximise the chance of capturing *Leishmania* antigens. After each elution, the dishes were washed 10 times with PBS prior to re-use.

Eluates containing captured proteins were pooled into two volumes: one from Dish 8, the other from Dish 35. These were buffer exchanged into PBS and spin concentrated at 5°C in 3 kDa molecular weight cut-off (MWCO) Amicon Ultra filters (UFC500324, Millipore) at 14,000 *x g* in several 20 minute spins until the total volume of each was reduced to 100 μl.

### Sudanese VL urine

#### Concentration of urine proteins

Sudanese VL patients’ urine from seven individuals was pooled, 0.4 ml from each to give 2.8 ml, and spin concentrated to 190 μl in Amicon Ultra 3 kDa MWCO tubes, with a final protein concentration of 12.5 mg/ml (hereafter ‘VLu’ for VL urine). Pooled EHC urine, 0.5 ml each from 14 individuals, was spin concentrated as above, down to about 100 μl (EHCu). Both urine concentrates were then washed to remove salts by making up to 0.5 ml with PBS and spinning down five times. These two concentrates, VLu and EHCu, were run in SDS-PAGE as described further below (Fig 1, upper right).

#### Affinity chromatography

Separately, another pool of Sudanese VL urine comprising 0.5 ml each of 57 individual urines with 1/100 protease inhibitor (P8340, Sigma-Aldrich) was concentrated with a ProteoSpin Urine Protein Concentration Micro Kit (17400, Norgen Biotek Corp) as per manufacturer’s instructions and in batches to avoid overloading the capacity of the kit to a final volume of 3.7 ml. To capture *L*. *donovani* antigens in urine, rabbit anti-DD8 was coupled to a cyanogen bromide-activated Sepharose matrix (C9142, Sigma-Aldrich) following manufacturer’s instructions and the 1.75 ml of gel was loaded into a disposable column. Due to limited amounts of anti-LV9 and anti-1S2D antibodies, anti DD8 was used for chromatography. The column was equilibrated using ten column volumes of PBS prior to use. Urine protein concentrate was incubated on the anti-DD8 column for 10 minutes, drained, washed 3 times with 10 ml PBS and bound material was eluted in 8 x 1 ml volumes of 0.1 M glycine pH 2.7, neutralised with 32 μl neutralising buffer per 1 ml eluate. Eluted fractions indicated to contain antigen by a dot blot were spin concentrated to give ‘VL urine antigen (uAg)’. Briefly, the dot blot consisted of 2 μl of each fraction dried onto nitrocellulose, blocked with PBS + 3% w/v non-fat milk powder (Premier International Foods, Spalding, UK) (PBSM), probed with anti-DD8 and detected with anti-rabbit IgG-horseradish peroxidase (HRP) (711-035-152, Jackson Immunoresearch) and DAB/CN substrate, with details as described below.

#### SDS-PAGE and western blotting

Both of the Sudanese VL urine products, namely the urine concentrate (VLu) and immune-selected urine antigen (uAg), were subjected to non-reducing SDS-PAGE in duplicate lanes of a 10% acrylamide Tricine gel as described by Schägger [25]. Half of the gel was stained firstly for carbohydrate using Pro Q Emerald 300 (P21857, ThermoFisher Scientific) visualised at 280 nm (Gel Doc, ThermoFisher Scientific) then the same gel was stained for protein using Sypro Ruby (S12000, ThermoFisher Scientific) and visualised with 488 nm excitation and 610 nm emission wavelengths (Typhoon Trio phosphorimager, Amersham Biosciences).

The other identical gel half was transferred onto 0.2 μm pore size nitrocellulose (10600015, GE Healthcare) at 160 mA for 120 minutes and air-dried. The membrane was then blocked using PBSM overnight at 4°C then washed in PBST, three times for 10 min each. Rabbit anti-DD8 and anti-LV9 were conjugated to HRP using a Lightning Link kit (701–0000, Expedeon) then diluted together at 1:400 and 1:500 respectively in PBST + 3% milk powder (PBSTM) and incubated on the blot for 2 hours with gentle agitation. The membrane was washed with PBST six times for 5 minutes each before addition of DAB/CN substrate solution (30 μg 4-chloro-1-naphthol dissolved in 5 ml methanol then added to 40 ml PBS, followed by the addition of 10 μg of 3,3’-diaminobenzidine dissolved in 5 ml methanol and finally 15 μl of 30% H_2_O_2_). The blot was incubated for 15 mins with gentle agitation in the dark before stopping the reaction by washing in deionised water several times.

Due to limited signal gained from these rabbit polyclonals conjugated directly to HRP, the blot was stripped using stripping buffer (0.2 M glycine, 0.1% SDS, 1% Tween 20, pH 2.2) for 10 min with agitation, followed by 2 x 10 min washes in PBS and 2 x 5 min washes in Tris buffered saline with Tween (20 mM Tris, 150 mM NaCl, 0.05% Tween 20, pH 7.6) (TBST). After stripping away the previous reaction, the blot was re-blocked and probed with the same two rabbit anti-*L*. *donovani* antibodies without HRP conjugation, followed by anti-rabbit IgG-HRP at 1:1000 and DAB/CN substrate as before. Bands in the carbohydrate/protein-stained gel half, corresponding to immunoreactive blot bands in the two VL urine sample types, were excised for mass spectrometry.

### Mass spectrometry

The excised gel bands from Sudanese VLu and uAg, as well as the two Indian VL urine eluates from the panning dishes, were submitted to trypsin digest and liquid chromatography tandem mass spectrometry (LC-MS/MS) at the Advanced Proteomics Facility, Oxford University, UK.

For all samples, mass spectra were assigned to *L*. *donovani* peptides by a simultaneous search using Mascot (Matrix Science, UK) against both the *L*. *donovani* BPK282A1 reference [26] and UniProt *Homo sapiens* protein databases, which contained all the proteins of these organisms, as deduced from their genomes [27]. Criteria for identifying *Leishmania* peptides were being rank 1 (the peptide with the best match for a particular mass spectrum) and having a ppm of -10 to 10 (error on experimental peptide mass values, fraction expressed as parts per million). Proteins were identified when they contained 2 or more different peptides matching the above criteria and were taken forward to further analysis (Fig 1, lower panel). Once identified, peptides and proteins from VLu and uAg from Sudanese samples were considered together for subsequent analysis of their properties, as detailed below.

### Epitope prediction

Epitope prediction was carried out to indicate short, *Leishmania*-specific peptides within the identified proteins that may be more easily synthesised for proceeding with antibody generation and test development. The complete amino acid sequences of proteins identified by 2 or more peptides were obtained through UniProtKB [27] and submitted to B-cell epitope prediction by BepiPred 2.0 [28]. The epitope score threshold was set at 0.65 (on a scale of 0 to 1), which gave a specificity of 99% and sensitivity of 2%, with an alternative threshold of 0.55 for higher sensitivity (specificity 81.6%, sensitivity 29%). Minimum length was 8 residues, with no maximum.

### Specificity of proteins and epitopes to *L*. *donovani*

Specificity of the mass spectrometry-identified peptides to *Leishmania* was ensured by a simultaneous search of the data against both human and *Leishmania* proteomes, with only those matching to *Leishmania* being taken forward to ensure that they were not chance matches to human peptides. This was followed by sequence analysis to confirm genus specificity. Proteins, and predicted epitope peptides from these proteins, were assessed for sequence similarity to other species using a BLASTP search against the NCBI non-redundant (nr) database with no species restriction and using the default settings. For epitope sequences, the BLASTP search was optimised for short sequences. BLAST output was assessed for the sequence identity between the query and biologically-relevant species, i.e., human pathogens and commensals. Sequence similarity to other *Leishmania* species was tolerated if *L*. *donovani* was a complete match.

### Signal peptide prediction

Proteins were submitted to SignalP 4.1 [29] to predict the presence of a signal peptide, which provided evidence that the protein may be secreted from the cell by this method, although *Leishmania* also has other secretory pathways. SignalP 4.1 is optimised to distinguish between transmembrane domains and signal peptides [29].

### Glycosylation prediction

N-linked glycosylation of proteins was predicted using NetNGlyc [30], which identified potential glycosylation sites via the R-group nitrogen atom of asparagine in the Asn-X-Ser/Thr motif, where X could be any amino acid residue except Pro. Glycosylation could indicate that the protein sequence underlying the glycan is inaccessible to capture antibodies, or that the glycan itself could be investigated as a diagnostic antigen.

### Protein properties

The possible identities of hypothetical proteins identified by mass spectrometry were sought using online tools that identify domains and protein features based on sequence similarity with known proteins: NCBI domain enhanced lookup time (DELTA) BLAST tool [31]; InterPro [32]; BlastKOALA (KEGG Orthology and Links Annotation) via KEGG [33]. Additional information on protein features was from Expasy Prosite [34] and published literature.

## Results

Using mass spectrometry, we identified four *L*. *donovani* proteins in Indian VL urine and two in Sudanese VL urine. Epitope prediction returned 33 B-cell epitopes of 8 or more amino acids, within the complete protein sequences of the 6 proteins. Of the 33, 20 had complete identity only with *Leishmania* spp. in a BLAST search of the NCBI nr database of all organisms. Details of peptides and proteins from each urine source are described further below. Full protein sequences, annotated with the detected peptides and predicted epitopes, are shown in S1 Fig.

### Indian VL urine antigen capture

In total from Indian VL urine, 19 different peptides were identified as those of *L*. *donovani* after a search of the mass spectra against *L*. *donovani* and human protein databases simultaneously; 11 and 12 from each panning dish respectively, with four present in both dish eluates (S1 Table).

Four proteins, one of which occurred on both panning dishes, were confidently identified by more than one peptide (Table 1). In addition to proteins, 10 solo peptides (i.e., the only representatives of their parent protein) were identified with a highly reliable ID by mass spectrometry and high specificity to *L*. *donovani* (S2 Table).

**Table 1:** *Leishmania donovani* proteins identified by more than one peptide in urine of Indian and Sudanese visceral leishmaniasis patients.

Sample	Origin of peptide	*Leishmania* peptides identified by MS	Parent protein (UniProtKB/GenBank)	Predicted epitope sequences with high specificity to *Leishmania* (aa length)
Indian VL urine	Dish 8	EYEELR; ALAEGQER; AKAEAEAAR	Hypothetical protein (LdBPK_191140 / XP_003860289.1)	VDDRTHREA [9]
				QRQRQHAHA [9]
				RRQRHTSP [8]
				RNRPESSH [8]
	Dishes 8 & 35	LSRSMEVR; LSSVQAGEVR	40S ribosomal protein S9 (LdBPK_070760 / XP_003865205.1)	SSRRASTTKPGPPPRAS [17]
				GMQLVGELNDSLD [13]
				LDQQPSVGTTT [11]
	Dish 35	FLDKLR; RSSQSSTSATYR	Hypothetical protein (LdBPK_323250 / XP_003863736.1)	SDNGASPGSRSPRSSRRSSQSSTS [24]
				SPAHQRSRAGASRSASRQG [19]
				STKRPRQSAVYG [12]
	Dish 35	ALISPSVLR; LSDAPRVCR	Protein kinase (LdBPK_262110 / XP_003861796.1)	NSSSYSGSLGSPAS [14]
				VSPVRRNSSSTAL [13]
				ANGGNSSSNSYT [12]
				QQQQQSSNRPS [11]
				AGTARLGSSS [10]
				RSTPRAGMP [9]
Sudanese VL urine	VLu	EFVVSGAALR; ITDMQREIR	Hypothetical protein (LdBPK_160110 / XP_003859699.1)	VRFRPNASLADGDAKSSAHGTVTQYGSPA^‡^ [29]
	VLu	ITSDEVLR; TVNEDLSR	Protein kinase (LdBPK_351070 / XP_003864692.1)	ANDDSESATRVEGLQVMSDINSIPL [25]
				DGQQIKVSSSGGGSSSKGSSNSTGS [25]
				KEERQRMHA [9]

VLu: concentrated proteins from VL patient urine; aa: amino acid.

^‡^ This peptide was identified by the lower epitope score threshold of 0.55 because the protein did not contain epitopes >8 residues at a threshold of 0.65.

### Sudanese VL urine antigen capture

**Fig 2:**
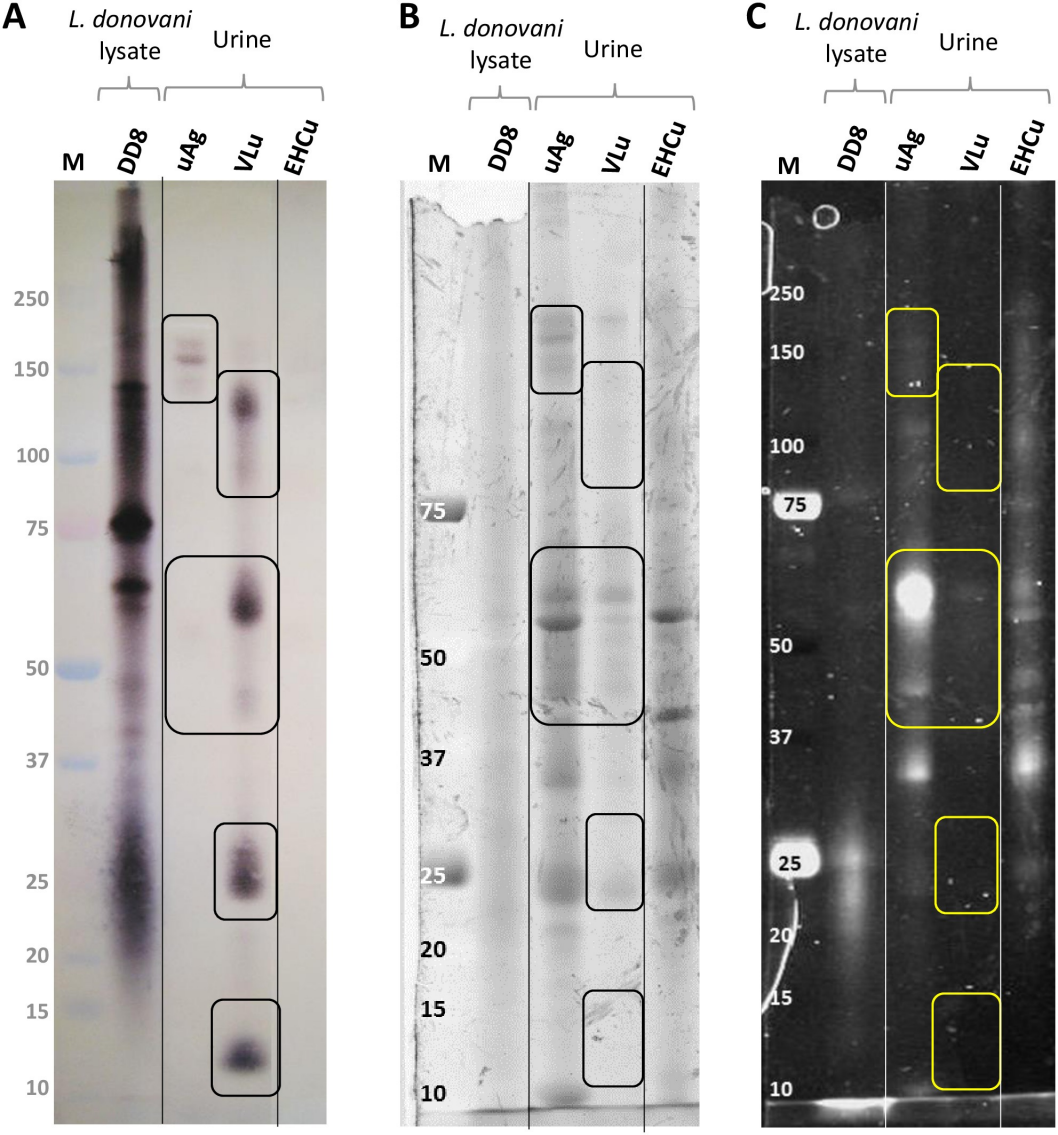
Sudanese VL patients’ urine (VLu) and immunocaptured urine antigen (uAg) detected by rabbit anti-*L*. *donovani* DD8 and -LV9 by western blot. Regions submitted to mass spectrometry are broadly indicated by boxes. A) western blot, B) corresponding gel stained for proteins, C) the same gel as B, stained for carbohydrates. Molecular mass marker (M) sizes in kiloDaltons. Lanes are: lysate antigen of *L*. *donovani* strain DD8; uAg, VL urine material eluted from an anti-*L*. *donovani* DD8 immunochromatography column; VLu, concentrated VL urine; EHCu, concentrated urine from endemic healthy controls. Vertical lines indicate where the photographs were spliced to remove non-relevant lanes from the original images, and realigned based on the molecular weight marker.

*L*. *donovani* peptides and proteins were identified by mass spectrometry of excised gel bands corresponding to immunocaptured urine antigen (uAg) and immunoreactive VL urine components (VLu) (Fig 2). Protein and carbohydrate staining of the SDS PAGE gel revealed that very few of these antigens were glycoproteins (Fig 2B and 2C).

Mass spectrometry of the Sudanese VL urine material (uAg and VLu) excised from the SDS PAGE gel confidently (>1 peptide) identified two proteins and nine additional solo peptides (Table 1 and S2 Table). The peptides with a confident mass spectrometry ID had complete identity only to proteins from *Leishmania* genus according to a BLAST search, but part of some sequences occurred in other human pathogens or commensals. Specificity to *Leishmania* was improved when two peptides from the same protein were considered together.

### Specificity of proteins to *L*. *donovani*

In total, we identified six proteins with confidence (>1 peptide) from Indian (4 proteins) and Sudanese (2 proteins) VL urine that showed high specificity to *Leishmania* spp., although not necessarily to *L*. *donovani* (Table 1). From Indian VL urine, protein kinase (LdBPK_262110) and hypothetical proteins (LdBPK_191140, LdBPK_323250) showed very high sequence similarity to several *Leishmania* species including *L*. *infantum* and species causing cutaneous leishmaniasis, and moderate identity to *Trypanosoma cruzi* proteins. The 40S ribosomal protein S9 (LdBPK_070760) also had high sequence identity to that of other *Leishmania* species, but little to any other genera.

The two *L*. *donovani* proteins identified in Sudanese VL urine, hypothetical protein (LdBPK_160110) and protein kinase (LdBPK_351070), also had very high homology to *L*. *infantum* with minor sequence differences between these and other *Leishmania* species. Moderate homology was found between the hypothetical protein and those of *T*. *cruzi*, whereas the protein kinase had high homology with *T*. *cruzi* proteins.

In addition to the proteins, 19 solo peptides were identified in total by all methods, 10 from Indian and 9 from Sudanese VL urine (S2 Table). Seven of these were from named proteins and all others were from hypothetical proteins. Interestingly, there was no overlap between *L*. *donovani* peptides from Indian and Sudanese VL urine.

### Epitope prediction

All 6 *Leishmania* proteins identified in the Indian and Sudanese VL urine were submitted to epitope prediction using BepiPred 2.0 and together contained 33 peptides at a score threshold of 0.65 which optimised specificity and 119 epitopes at a threshold score of 0.55, which provided higher sensitivity (S3 Table). None of the 6 proteins contained signal peptides and 4 contained potential N-linked glycosylation sites (S3 Table).

### Potential VL urine antigens

The 33 epitope peptides predicted with high specificity within the identified *Leishmania* proteins, and one additional with lower specificity, were assessed for specificity to *L*. *donovani*. Twenty of the 34 epitope sequences were selected as they had complete sequence identity to *L*. *donovani* and little or no identity to sequences from other relevant species *i*.*e*., human pathogens or commensals. Specific peptides or possible epitope regions indicated for production of antibodies for use in antigen capture assays are detailed in Table 1.

## Discussion

Validated tests for urine antigen in infectious diseases include those for schistosomiasis [35], tuberculosis [36], *Legionella pneumophila* and *Streptococcus pneumoniae* [37], among others. Here, we captured *L*. *donovani* antigens from VL patient urine by two methods using immobilised anti-*L*. *donovani* antibodies in panning and chromatography. Both methods yielded parasite peptides which we identified by mass spectrometry. These led to identification of six *L*. *donovani* proteins from urine of VL patients from India and Sudan. The proteins were predicted to contain highly species-specific epitopes that therefore make good candidates for targets of a non-invasive urine antigen capture immunoassay for VL. We additionally identified 19 single peptides with very high identity to *L*. *donovani*, which provide evidence of additional parasite proteins in VL urine.

Studies using similar methods to identify pathogen antigens in urine include those of Abeijon et al. [22, 38] who identified *L*. *infantum* proteins tryparedoxin peroxidase, superoxide dismutase and nuclear transport factor 2, by mass spectrometry of concentrated Brazilian VL urine excised from gel bands, as well as *L*. *donovani* proteins encoded by genes *Ld-mao1*, *Ld-ppi1*, *Ld-clp1* and *Ld-mad1* from Indian and Kenyan VL urine. We did not identify any of these proteins, and in addition, none of the peptides and proteins that we found occurred in both Indian and Sudanese samples. This suggests that there could be many proteins or protein fragments of parasite origin in the urine of VL patients. However, differences between the studies were that we used an antibody (1S2D) to capture antigens while other studies did not. We searched mass results against a single genome database to identify peptides, which may have limited our findings, whereas Abeijon *et al* used a wide range of genomes [38]. However, we performed a rigorous quality control in order to exclude false peptide matches to *Leishmania* of peptides that were actually of human origin.

The proteins identified here are likely to be intracellular, based on their identities and features. However, *Leishmania* has various secretion pathways, therefore the proteins may be secreted by non-classical mechanisms [39]. Four proteins did contain potential glycosylation sites, a feature more common to surface proteins involved directly in host-parasite interactions [40, 41]. The carbohydrate and protein staining of the SDS-PAGE gel also indicated that very few proteins were glycosylated, and those that were, were not detected by antibodies on the corresponding blot. This may be expected as the rabbit antibodies were raised against a soluble lysate antigen from a preparation method that favours intracellular contents rather than cell membranes. By comparison, the KAtex assay that detects a carbohydrate antigen uses an antibody raised against whole intact parasites [13].

Three of the 6 proteins identified in VL urine were hypothetical proteins, defined by the presence of start and stop codons in their genomic sequences but without experimental evidence for the protein itself. These proteins were submitted to several protein domain identification tools to reveal possible similarities to well characterised proteins and elucidate their features and possible functions in *Leishmania* (Table 2).

**Table 2:** Functions of *Leishmania donovani* proteins identified in urine of Indian and Sudanese patients with visceral leishmaniasis.

VL urine origin	Protein accession number	Features	Functions
India	LdBPK_191140^a^	Predicted zinc finger RING-type domain; predicted coil regions	Ubiquitination pathway and other intracellular protein processing pathways.
India	LdBPK_323250^a^	DENN domain	Involved in GTP/GDP exchange and occur in other proteins that regulate membrane traffic in eukaryotes.
India	LdBPK_070760	40S ribosomal S9 protein	Protein subunit of the 40S ribosomal subunit.
India	LdBPK_262110	Protein kinase	Add phosphate groups in cell signalling pathways.
Sudan	LdBPK_351070	Protein kinase	Add phosphate groups in cell signalling pathways.
Sudan	LdBPK_160110^a^	MORN repeat motif	Unknown.

^a^ hypothetical protein.

The great difference in western blot and SDS-PAGE gel profiles between the concentrated Sudanese VL urine (VLu) and urine antigens (uAg) purified from the same sample type was unexpected. However, the sensitivity of antibodies used may detect proteins that are not readily visible on the gel. In addition, the process of stripping and re-probing the blot could have somewhat altered the composition of the blot. However, the EHCu remained negative, indicating very little non-specific reaction. Heating the urine, which we did not do, could have led to more specific reactions [42], however, this favours carbohydrate antigens as it denatures proteins and we sought to retain as many of the conformational protein epitopes as possible by running non-reduced samples on gels.

Coiled protein regions are often made by repeats of a few of the same amino acids, as identified here in hypothetical protein LdBPK_191140, and this repetition can lead to high antigenicity, making them good targets for immunodiagnostics [43–45]. The geographic overlap of VL with Chagas disease, caused by *T*. *cruzi*, particularly in Brazil, makes it imperative to identify a *Leishmania* antigen that will not cross-react with this other trypanosomatid. Although the proteins identified here had very high sequence identity with *L*. *donovani* or *L*. *infantum*, some had moderate and one had high homology with *T*. *cruzi* proteins. Therefore, a polyclonal antibody against a complete *L*. *donovani* protein could also react with *T*. *cruzi* proteins. For this reason, as done here, selecting shorter and more species-specific peptide sequences is preferable because these contain fewer epitopes and generate a more specific antibody response, although it is unclear whether these epitopes would be linear or conformational.

Based on the species specificity of the epitope regions, we suggest that combining several of these, either for raising a mixed polyclonal antiserum, or later combining individual antibodies in the assay, may improve specificity and sensitivity of the prospective assay. This was found by Abeijon et al. [22, 46] where combined assays had improved performance over individual analyte assays.

A strength of our study was that the criteria for identifying parasite proteins in VL urine took into account the large number and amount of human proteins in the sample [47]. Initial steps to enrich for *Leishmania* antigens using specific antibodies assisted in achieving peptide identification by mass spectrometry. Further, by searching mass data simultaneously against both human and *L*. *donovani* protein databases, we were able to exclude mass spectra that had a better match to human peptide sequences, thus excluding potential false positive matches to *L*. *donovani*. The dual searching did not exclude possible matches to bacterial peptides, therefore we searched both the peptides and the proteins against the NCBI nr database to exclude this possibility.

A potential limitation to our technique is that while we have presented *L*. *donovani* protein identities here, there is no certainty that these proteins exist as whole proteins in VL urine as we can only identify short peptides by mass spectrometry. However, the presence of the peptides suggests that at least fragments of the proteins do occur in urine, along with many host-derived proteins [47, 48]. We used a large volume of urine from multiple VL patients in order to capture the peptides that were identified. However, with more abundant samples, it would have been beneficial to conduct replicates to investigate the frequency of these peptides in VL urine. Progression from identifying VL urinary antigens to having a prototype rapid diagnostic test (RDT) relies on follow-up to synthesise antigens and raise antibodies. An alternative method to develop monoclonal antibodies directly against precipitated urine proteins could also be attempted, followed by screening for sensitivity and specificity for VL, as used for malaria [49].

## Conclusions

We used various methods to capture *L*. *donovani* antigens from VL urine from India and Sudan, including panning of urine proteins with immobilised anti-*L*. *donovani* antibodies and visualisation of immunoreactive bands in VL urine on a western blot. All methods yielded *L*. *donovani* proteins by mass spectrometry of captured or immunoreactive material and 5 of 6 proteins were highly specific to *Leishmania*. In addition, epitope prediction revealed 20 *Leishmania*-specific B-cell epitopes that we present here, that make ideal candidates for synthesis and to generate antiserum for antigen capture assay development.

## Supporting information

S1 Rawimages (PDF)

S1 Fig Amino acid sequences of *Leishmania donovani* proteins identified in urine of visceral leishmaniasis patients. Bold, underlined text indicates peptides identified in urine by mass spectrometry. Boxed text indicates predicted epitopes within each protein.
(PDF)

S1 Table Number of *L*. *donovani* peptides and proteins identified by mass spectrometry of antigens captured from Indian VL urine with anti-1S2D antibody. (PDF)

S2 Table Solo peptides of *L*. *donovani* identified in urine of Indian and Sudanese patients with visceral leishmaniasis. (PDF)

S3 Table Number of epitope peptides of ≥8 amino acids in *L*. *donovani* proteins identified from VL patients’ urine. (PDF)
