# Supplementary material for: Isolation and characterisation of Leishmania donovani protein antigens from urine of visceral leishmaniasis patients
Source: PLoS One. 2020 Sep 14;15(9):e0238840. doi: 10.1371/journal.pone.0238840 (PMC7489519; doi:10.1371/journal.pone.0238840)
Supplement: S1 Rawimages — (PDF) [file pone.0238840.s001.pdf]

Raw images corresponding to Figure 2.

M: molecular weight marker
1: *Leishmania donovani* DD8 lysate
2: *L. donovani* LV9 lysate
3: Concentrated Sudanese VL urine
4: 'Urine antigen' as described in the manuscript text
5: sample not used in the present analysis
6: sample not used in the present analysis
7: Concentrated Sudanese EHC urine
8: sample not used in the present analysis
9: Commercially available human urine proteins not used in the present analysis

X indicates samples not used in the present analysis of VL urine.

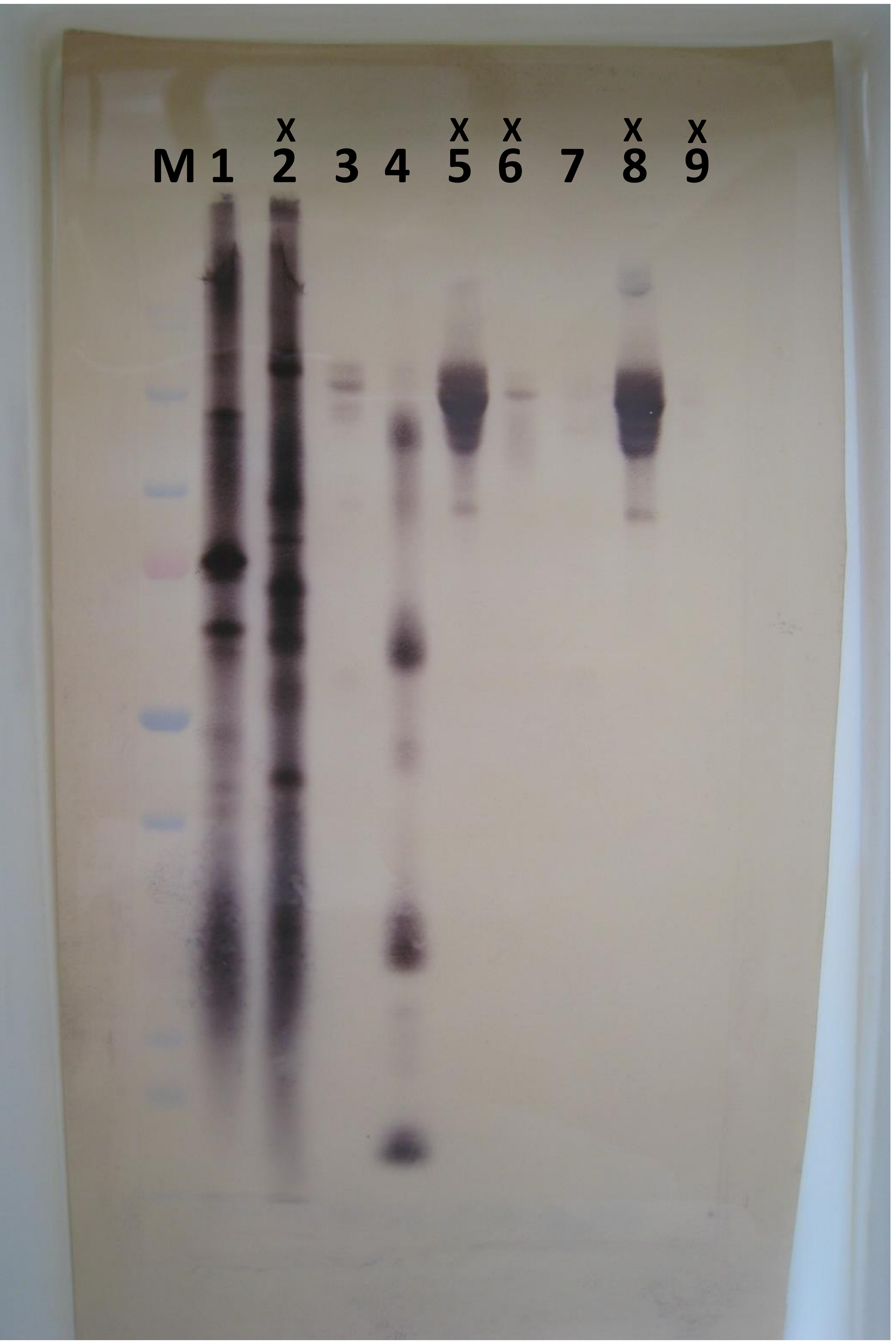

Blot photographed with Canon Ixus 860 IS digital camera.

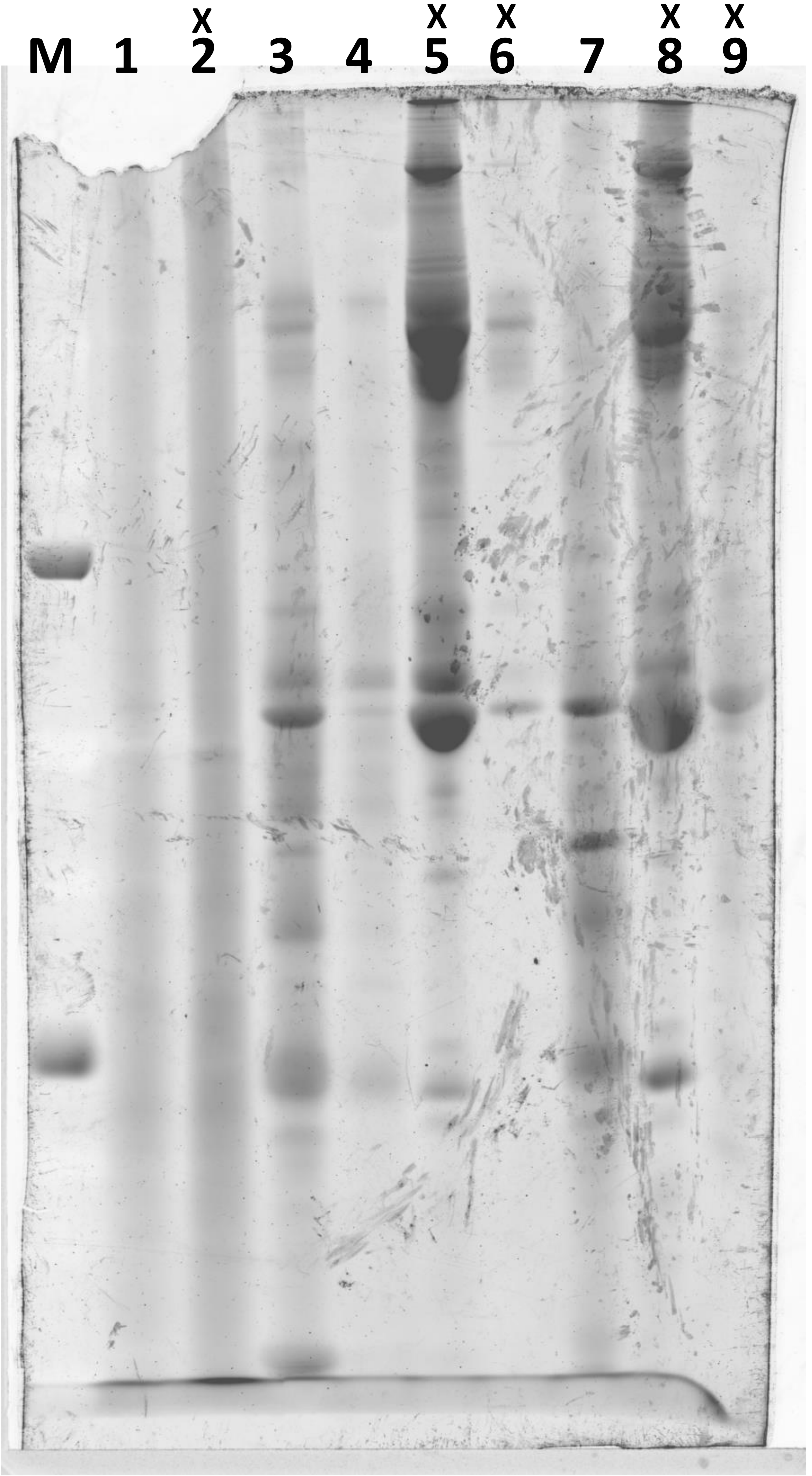

Sypro (protein) stained gel imaged with a Typhoon Trio imager.

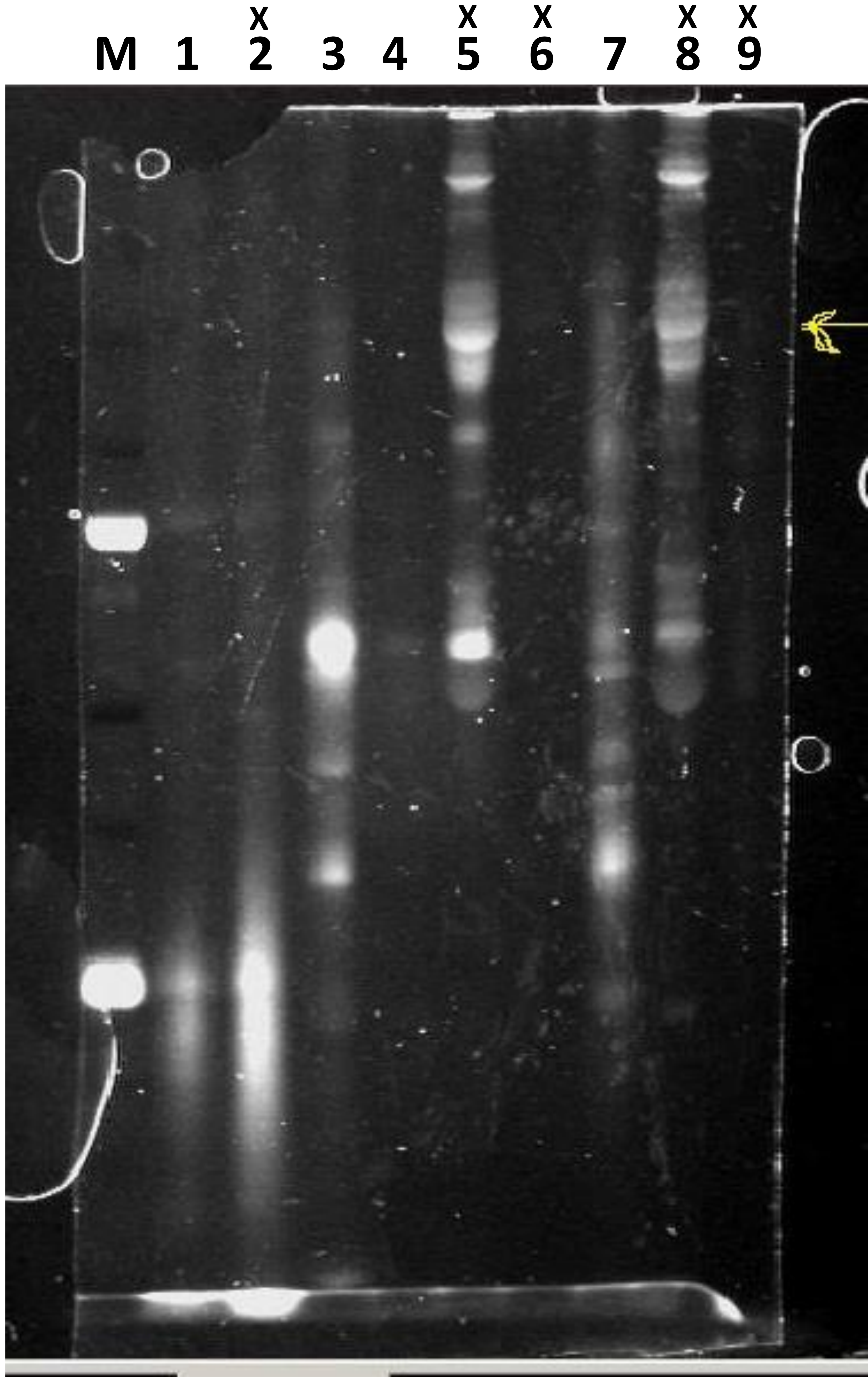

Pro Q Emerald (carbohydrate) stained gel- screenshot of image taken with GeneSnap software in a GelDoc imager.
