# Supplementary material for: Isolation and characterisation of Leishmania donovani protein antigens from urine of visceral leishmaniasis patients
Source: PLoS One. 2020 Sep 14;15(9):e0238840. doi: 10.1371/journal.pone.0238840 (PMC7489519; doi:10.1371/journal.pone.0238840)
Supplement: S1 Fig — Bold, underlined text indicates peptides identified in urine by mass spectrometry. Boxed text indicates predicted epitopes within each protein. (PDF) [file pone.0238840.s002.pdf]

S1 Fig. Amino acid sequences of *Leishmania donovani* proteins identified in urine of visceral leishmaniasis patients. Bold, underlined text indicates peptides identified in urine by mass spectrometry. Boxed text indicates predicted epitopes within each protein.

Indian VL urine

Hypothetical protein (LdBPK_191140 / XP_003860289.1)

MAGLQSENISINSTRLPEQSTSDKDICVFCLRRATSSASDPHAPLFPFFSLVDCRHYACQPCALVHCDNAGRRIFCPKCHCVSRLAQSGRRRTRSAAAATDADADR
VSIDDGVSSTRSRRTRTGTTPHRSALKGKSGTLKRRASSVQFPANPTTSIVPGDGVSVASGAVAVPSEGDSAGEQHHQRRASSPLTQEAVDALPLDPVEARRRRA
REQQQQLRAAEAAAKAEKAVGLYAITAAPPPKVAPHPPSATAASGLATNEGFKKVKDRSRSQPVPKLPHTILEPKEEYPVPPPLVLHTVEEEEAHAPAATGESGR
DAKPVLPAAAAGATAAATAEEDVKAEKSASALAPAWKTSVTPRQMEDEVEAAMAASADKSQTTGVLPPPTGTPEELARPHQLLDDCENTEAEERGVVGDLEA
HERAALMQRMEEEDTAIRARRGLVVEFDSTPILSQQQHPTLQNQHSHHDNTGSISLVDFSDQVSVTSDDELSQRASGYKSATRSDSASRRRRRTSVNGASELRA
QSHENMAGESDARPEKGGQAAAARTPGDALSSAAKSVAGSGSAEITLSPMTAARQGRVLASEEVDDGGKLIATRAALAEADAK**AKAEAEAAR**AAAEAEAHAE
AIRTAEEAR**ALAEGQER**ARQRQLQREAVEEAAEQKRQQQRQEEEVLRQRQQHEERAAFACQALQDREGLAREALEQAEAAVREHYERGADEWLTAALPHELE
AQRLVRAAAERRRQQEQQQAHLAAEEGLDRDEVEEAEANAWSWLLSGARVDRVVAATEEGDRVQR**EYEELR**FERLQLQLREDTAELVHEEAHGRASFIEYEA
ATRSLLQSHHAVQRQALAEGERRQEVELLKKAAARREAALRQRFQWELDELDAEEVSGRAMLREVEREERRTAHLVFLQTLASIRRKEVLAQHAAAVAAAAAA
DEPGGTSVLSEASTMIAAQVHRRVQQRQTDAAVPLMIEGDGDGVDDNTADMVGAATPPQFRTPLITAKELAELRAREASYAAELQAALERLREAERRVAEEAA
IRAQAEQERQAAHAESQRLLQEAEQRAEQRIREARDAAEQLLQAQLADLRDEAVRRAEHAAVMQALAEEEQRAVLEAKLQAAQRQLDEAQQRAQEEVRRAR
ADAAEMAAADAARAAREAQRRDEEWQERCRAEHLLRLAEQEREKEEAVRRLAEIEVRAAEAVRLAREEAARTAAEAQERLAEMERRQQAREAEVQSAAQRV
RSVRDSLREFDSSTQSSRYATPFRAVLPGQRPPTSSSSYRAPSLVAASSTHATHTAGAVATQASVHTPLQTPPLVPSSSRTSSAVRRQPSAQSPTPRVAARVPAAG
AAASLAPSDATPPKVIISSQHTPLSSAVAPGLGRSGAAVPSSSASAAGATSLAAMDPADIIRAAIRSAVQEIVEAQRRTQTLQDRAEQNVELSERRYHRRQSDRLR
AARDVAAIESSYAASEVDESEERHQRWRAWSQQPHQRTRQRQPVRGYERDSRVASSRMSEERLRSVRSSRGDYAQEVSESPWTRSSSSPASRLSPTGAPLSTA
PPAPAARSVSSNTLLSTSSVYFDNSYQPSAAAFGAAHQHFRRNGCDYAAVQQQQQQQQRLASFAPYSSASRVLFAPRVAHQAREAWAAEDGECGYAQRMH
AGVGARAYSRVASEQQQRYATGAAQPPPRQPLLQTVGVAAALPSAAHAYNEPEPLPPAPAAMEYSPPPRRSTTRAAAEVVPQQQSRPAGVGANVGSGGGIA
ALPEPSRMCPRCYRTDTTSPCWRCGEMICRHCGLPPGSARKLCCTAHHRAQLREFTRRKTYESGKAATTVGAGKVPSPPPPQQQQQQQQQQRLRGEQQVQT
SFASSAASARSHDAAHFADEPAARLDVGTSPLTSISPPSMISPSAYRAPEQRQRQQQQPQAQPHYPSSYQAFTMAGPAAPSQQPIYSPGYHSAATTYPYAYSHP
MSQPHQLPTHQLPFYADSERPHPMPPPLDPYVQPFGQPQPYAPFGYSVRAATAALGSAATGADFAAPMTVSEAQRQQGTTAVAAEAWATHEVPPPESRAA
AFGAVARHHASPAELTQSPPPPSALPPSQQEGASEVMGLRAAEGGAMIETVAPAAAAVDDTPDAQVQRRPSTSSWVDDRTHREAEQTVAAENRTLPNATVA
AAVAGSAAADASLPDSAPEKQGGTTAPVDARSTPDGGAAAENDGDGTSAATAGATAAAVLEEPKTERKKTFPAFFVSLGDGDDAAEPRRPKPPTPRNFVPSQL
APPTPPRKQQRRTLPSGMLLTSQNRSGRQSAHHQHQCRKLSPLQAHETNCARNSRKSVSPCRGFGAHRRQRHTSPSPYEQVPKASGARDGPRKTPSHPSSKQ
RQRQHAHANGTATSITKSIKSYIKSLSPMVVVDESGTPVVYEDVRKVESNNPWMQQQPQRPQGWSATATPAAHDIYLSSGGIRSVGAEAERNRPESSHGHSK
MGRYYVNVPSQPHDSSPQKHQHHQHQRSSHSDSYYPYFTSSGATSETQSQPPQQQLQQHLYAPPQSHIQTDITYAAPFVDRRVPTLAELEKRLQQLRIVDEYEA
AQYHRRQAQRAHQQQQQRIHVLLHSPGAVAAGGNTGAFVTASSWATRELAQPLQRRHSSATGRHPQQRTVSPPWRTDLNSSPVRPRWDISQPRSPIYHPAP
ATMTGN

40S ribosomal protein S9 (LdBPK_070760 / XP_003865205.1)

MPTSSQASASSRRASTTKPGPPPRASSAAPPLPARFPSHSQAPLPSATTSRRGSAIPELIGTRRDSSIGATSTNPRLRGGSVIDAAAAQRSSSAPRNPIDFASKESQE
AMNRLRRLFLPVMMRVILRFRRRSRWKDGQPPRQYTPGSVVAAIMSTKSLLADCPQRMVESLAAGATFMSLTPKEIIVYANESHVSCGIVVLLYGQLEERRPEP
GGKKPNAGGGGGSTRPICTQPHRLHKAMSVLCLMPVMCEDRATSFLATRESEEADVAIISSRWFWEVTYSMAQASLTTADVMGRTLREVVLPHRRDLLLADYF
PTSVVLLRSWMWSMLTASDRVK**LSRSMEVR**VLSVGDVLFDEGDYCPYIYVVRRGALAAIVKGETLAVLEAGAAVGEESVLFHDRRNCRVVAITVCELYALHAHH
LLRRFLKYPESARRIVAAAIERQVWWMEEGRTRDVFGLVSILSGVPCLGHTTDAMRDEIARCASVLILPQGHTVVSANTPCTFFCVIGRGSVTLISSMKTAAVAET
PSSVNGIDSKGESQLGMRRSSAMKPMSNPATEMRRESRSAGDFFGELCLKPHLWPYDVMCDSTVSLWQFDREAVLGVLERNRADAQALEVCRQGIGLYRTQ
RGETSIIDGFEPPATPNAPANGWRSCIGGQRSRAQSTDFATAPPGARSSSAGSRAGRRLSLSHAAAPEWLSRGSPSEGRTPRGGRRAGVEWTPEQWTTYAMT
RLQDGLRAEDAKETPPAPSRELRGVDKEVEKTMNEKVLQLVAVQPETPPNPADCDISGDSSELQSIIVGQLFLIMTEKQSRFLRQVKDSNVRLITEDEGMQLVGE
LNDSLDDMSGMDALHNSEALVEVTEVMQLEDISASPLEGSHFLPQRAMPSLSVRDSTDATDYINVHRVDDALSVEGSGGALAGWPMPNASIGRTRRSTSVGA
LGEASGVLGASLNPRSSLNRSHFVRPSSAVPPSPRCASGRDDFLSPGAIGVVRPASSSEPRMMPPARTRPASVDFNQFNNKLSVDRSTSMLDRYVKIEDQNYFDS
FVKVLPLQRDEMWAPDGVNTDSEMGTGSMVLLLLHVRKCDYLSAKVMQRCARPIVKVTLGERVLVRTSVMENRTAPRWPIEHSSFISFVRRGTDIVFSVCDAG
DECRIVYQASFSTASIHENGGVEQRTMPLTELSVTGFPANAEMDADVKSVESVGRMQRSAKKPRMTMTMLAVTANKYKALRQYLETKEKVIVDPPGTPESTKL
FLQVMSVEGLKHRIEATVTASLYNGATSTTVLKTERVIPKTRSPAWPGDTSFVIISGEGGILSFDLHHRDAVIGSTETTVDELIFGGVGLRRLPLLQAQTGRLVIGHLV
VGVLGARLGDSVESRNRDWVTHLAVEELSLAREGFSINPDPFIVLRSGTGAELMRTPLAFSAFEASWSMSEASCLLQCPRLCGSTVSYQLEVCDSDEKEVIGRATIL
LSDRGLGPGHLHDLSLDPPGRGVVRLRSLCLPVLELPARGAAGKASSAAALCPPFSSHLHLSESATLLLLHMGGCTNLPGGATVELQIDAIGTLSVDAQPYLRTAV
QEATTALQWPLSKASVLLRIPSRGDVDDRAAEAVDLRQWQCGHQCHFAVYDGVVDDVSQIGQVAVPLSQLLNTALHTYPLFPRCMDADGGGLDQQPSVGTT
TRPRAAAERTLGNLQVFTLLGSLDHQVRGTAEQETDTLLTSVPAAGGHAPPDQLPYYNPESADITAARLASALPTTVVLSVSNICHVLPSASEKYVQIVVRRGATV
VLSVERQMGVLSHAEWSPTEASAVVSCGALPADAALVVELVAMDVVEGRSDEEEDGITPSAAGAKAESTARDKTRRRTITKGNASAANLPLGHAELPVSR**LSSV**
QAGEVRVVTLMLSRSQRSSSLSETTGRLPRVTPSMCALKDAQVVWPTVSFCIFGNRA

Hypothetical protein (LdBPK_323250 / XP_003863736.1)

MRQRTVSRSRARSQIRISAAEEDDLRVGLQR**FLDKLR**QHQPLFTEDDVTAISFPPEGSSSSSCVGGGSPHSSASTSPRHSPSRADNEEATLDGTFKGGGSSPSMAK
CRKVPAASPTTTGSPTFASASVIDLDVPLSAPLFLTAVNIFEAKLLSKSKRVSTDALKLVRQAYALVWRDPGYLATLMNGDPCLAGSSSTGSSTTPPDGWSRRPSSV
PLASSLSKPHLTCQALFSASAPAPTQRPDRRLALQLDECIRTLLVYANTSVNLCQSVSTNTRTLLLLAFVTCAARRLEEEDDVPYGDGPGSVADIVVGNRNLHAEM
KKLESLWRSCGIEGRVADYLRECYLVEFLLDAALPPAVCSARYDGLQMAVHEKKQRIISDVVASRAGFASMMRMAPLRGLCGLANMSAFGNSKLRARLLDKMA
SEGVVERLAAEMAAAMEAVLAPDTATTTPAHSSTKRPRQSAVYGGSPAHQRSRAGASRSASRQGSLDSVSPAGKERRAMTIARASTSLERLLDSDPTPVERIGQ
CVDILMLLTCPDLRPPDGGEESPSRSSTSSASAVVRSTTWASRDAALHEICLYLLAICFVSKTHARYAELSSLQLYAADCMLQCAAYSGAFYAMVVHAMDVLIDMI
ESMSAAPVASAQRTPLSSGRASCSRLVSPQKNWRKDAEEGDRLDSERGRRVLQHLLPGDEAGLLPLLTNLLQYTIHAQANAKSIRRWFGFLADRLLPEVFEAPQL
TTGRRSSATWGLQQQQEQRRRSQADSRASRLPGALGNGSPAAADGAFDAPVLARDLTRAEVVAAAPYFCFARRAVEETSWRDGTELLFAKVVLLAFHVIVTQG
HRCNLLFGQACACYDVVAPVLLLRSSQSDNGASPGSRSPRSSR**RSSQSSTSATYR**RSTLLMHLREDERASTQAPLHSNEEASEDGGVAGSEERETKGADEEKEVF
IGSTIRRRSLSTRQRQRGSAVKGSAASAERHLLATKAGEDDQGFSPFIQSPSRSLVFYEACSPSDTEASSDSPLDRRGSRRRRSSSSDRRSSDGGRSLGAALDEAAF
AAPTASATLAESPEAVTHSWTGRLSLRSLLSSIGGHSEAPLLDKEKRA

Protein kinase (LdBPK_262110 / XP_003861796.1)

MSNRSLHGGHTTIHNNNAYHDAAKEERQLQEQQKPSMPGSHAAVERMTAVQVVDRLRWWRTHRVREQPIPVDHFVLRGSYANYSSFGTRPLAPSVTRLLQK
ARTVRAQYLRTQQQPQHEAPRQSRQVSSLLMAPTAGASVRSASNERLGSDAGETERTTGCISEEEGQYGERQQRQLREMNDDAKEKVNNKRRAAVIASWPRS
PRPLNEVAIACLGEPAGQRDSATDNITRISLSASGSAGTSPPLVRAVSGSAPSVDTPSGALYVPSEQEARVAERLLMLFRPRAASAPLRSRRYRCTAGRLDATSSEFS
GEPPLQGDWGHAEATGLHHERMTRVSASDEITDVRRAPGAAEAVIVLHHAPRLVLYEKGLNPKAALRAAGISSDALESLDDAAAESSAELFALRNLPLFAFERFFY
QQQLRGLAETRRSHTPGMRSASADASAESAGVPESDTASAPTGAAAGAVAAADTAVDDDIIANGDDAFFFGSPLTATLACSLKTVVPLSSVAAAAAPGSRGRD
GRDAAARQAVQHRGRGRDDGSNRRRGGRAALRAVSVPALPPVYKGGCPQMTLATSHHHLSSGARLQQQQQSSNRPSPILSAGLRSSKAPQNGAHLCLRVDD
LQDVYVFNPVVDMIGKGAFSKVYAAVPILRGKEGLRRFASPLLDGQAAPCGSDELSGSGPREGGRRVSTVRRVSPVRRNSSSTALRVDPAAGAAVGDAAGPLTD
DETAKRRPHAQSLAASLHSIPVVALKVIPRKARQKPKAAPDPLATTHSTAAAAAGSVASNAQQAAQNDQNSVRRELVEIEREVSILRSLHHTGCSQFFEAIRTPDA
FVIAMRVFPGSMDAQRYLSRYGAPSEARAALLLFQLVSTVQYLHTNFGLIHRDIKLENILLSEADAGVPDARIREVLGPALHKSDTSAMMTEIEGGRKGCASATTA
AETRTAAQRRYSMLSRNVARLLRVTLIDFGLARRTRASTLSPIVAASRGRGAGAQHGSLNATITSPTHLASFPPTSASLAASHPHASHNPNSSSYSGSLGSPASSSA
GCAAGVVMLANGGNSSSNSYTPAKPAPPKPPLLCRPPSAAVGAGTARLGSSSAGISAMERSTPRAGMPSPMPSTANMFTRFLDLEEEMDEEENGGAAGTSN
TTANTTTKVGDLGRGGRRDMAENEDDSGAFSTDVSASETDCESEGGSTAEPKEGSAPNGEERLETGPQQEHVARK**ALISPSVLR**AHPQTPPGSAIIVSPPLPPTP
MLLSSTTAFTLDHFNAENSSSSCNTSGPAAGGAGLRNRCASCIYTSHQQLSPAPPVVAAGVSSAPDDTEATLLLTPCGTEKYLPPEVLSWILEHGWARRSTTVGLA
RAMDLYSIGIVAYVLLSGCFPFNASSRATLLQQQQRVPRCNSARWTGVSSAAISFVQRLLEPNPRKRMTAKEALEHPFLHEARQLAEKLSLVPHGEGEEASHPST
WRDGSHMDNHHRHSIGGTGRHFEDDANVHARSHWATSTTATTNLPHSAPSPTHPDHTHSGAAIERASTQPSSSVSLAVYDSQRRHAGGPCELAAANEPSPLS
SNSGADGGGELLCHHVESGTAPTATSISSGCR**LSDAPRVCR**SSDECERDDVRASVTRDVPGKVQPLLQVCKESPYSTTPPVPVTPTLAKPCTAALARAASIELPALS
SLVAHPAKPSPSSTLYFTATGTTASADGGRAERATLPEPESAAAGSSAPALAPGSKDAAAANRPVSLSPAATTSTARATEGGEDDLFESLYNNIMSSD

Sudanese VL urine

Hypothetical protein (LdBPK_160110 / XP_003859699.1)

MPIESAMSEAHTDFRGCSLLPDFAAVADVSERTGVGYLQYSNGAIYEGEWLNGERHGLGVCYYPSGNIFVGQFRSGLMEGPGTMFFATGECFSGEFRDSTIYKG
VYSRQGREICGVWKDGNRVSEMPTKAPEVLQRARAALFATIVEQVNEYLWGAASNPTKLVLSAPPEGAALKAPLDILSSSTDAHGTPKTPPQHLTVIPTFDAGN
QSFTSVRFRPNASLADGDAKSSAHGTVTQYGSPATVFACAAPAPNEIFSVRRFTLRCFVFLFPFLSLPWVPVAPLRITSLHEER**EFVVSGAALR**SDFDAPSFSMCVI
AVGMLCQIVAIILVASRVPLGKVQDGKLTLPEMVVPCVLWLLVALVKASYISFFRFPHGLERVDRRLTPKLSALAASLVDAKSSVCIFTYDDDGKGKVMNRHYKYR
WLLFGVVLGLLMSLSAPATRGGYGHNMFGSNGFQLTAALLAFFATFLFAATLTFSVLK**ITDMQREIR**AKLHVLTNLAFLERRCLMSPSKHASIAFDLDESFNAKNV
FSGFPGWYSLRSLILYASACANHRARTTAMRVFWFALVCAFIVGIADTLLMIQNNRPGDQYYSTAHSYAFFTFLFWGALLHRYLYVCISTRRELQHHLYIMDIAGL
YHRVKLSDSEAGDIIQQCRCISESNDAEPEIFPVIIYCFVVFLTVILNLAAMTAICYQLGRAIYR

Protein kinase (LdBPK_351070 / XP_003864692.1)

MTVLPPKINVPATPALDGVDINSLYDVNHGKLLGKGGFSEVLAVRHIPSGEIRALKVMMRPSLVGKKAEMVAHEKEILRRTCHPAIITLHEAVQTPDKVYFALDLM
NEDLFEFIVRNK**TVNEDLSR**AIMHQLMSGIAYLHEQSIVHRDIKPENILINVVVKSEANNAANDDSESATRVEGLQVMSDINSIPLEKLNVEVKIADFGLAKVVME
WDVCSTPCGTSFYIAPEVIRGIEEQGAKPLCTNQRLVKSVDVWSAGVVFYVLLCGRPPFHGQVRTGQDRRDLLRRIDHGVLFNPNHGWDSISAEAKNLILKMLD
QESSKR**ITSDEVLR**HPFFTAHGYSRPVPASDARRRFMQMQQLSLRTKVPAAQSAEAQVKTMSPLSGPDGQQIKVSSSGGGSSSKGSSNSTGSFLSSIKDFFGHRS
KHTSDISKEERQRMHAELAELQATVIAEEDQEGDVTSYKPSMPVKEAKPARTAVMNMKAKVGPDALRK
