# Supplementary material for: Isolation and characterisation of Leishmania donovani protein antigens from urine of visceral leishmaniasis patients
Source: PLoS One. 2020 Sep 14;15(9):e0238840. doi: 10.1371/journal.pone.0238840 (PMC7489519; doi:10.1371/journal.pone.0238840)
Supplement: S1 Table — (PDF) [file pone.0238840.s003.pdf]

S1 Table. Number of *L. donovani* peptides and proteins identified by mass spectrometry of antigens captured from Indian VL urine with anti-1S2D antibody.

<table>
<tr><th colspan="2">Indian VL urine
immuno-captured material</th><th>Dish 8</th><th>Dish 35</th><th>Found in
both dishes</th><th>Total
unique</th></tr>
<tr><td colspan="2">Total number of proteins</td><td>2</td><td>3</td><td>1</td><td>4</td></tr>
<tr><td colspan="2">Total number of peptides</td><td>11</td><td>12</td><td>4</td><td>19</td></tr>
<tr><td rowspan="2">Peptides which:</td><td>Led to protein IDs</td><td>5</td><td>6</td><td>2</td><td>9</td></tr>
<tr><td>Solo peptides</td><td>6</td><td>6</td><td>2</td><td>10</td></tr>
</table>
