# Supplementary material for: Isolation and characterisation of Leishmania donovani protein antigens from urine of visceral leishmaniasis patients
Source: PLoS One. 2020 Sep 14;15(9):e0238840. doi: 10.1371/journal.pone.0238840 (PMC7489519; doi:10.1371/journal.pone.0238840)
Supplement: S2 Table — (PDF) [file pone.0238840.s004.pdf]

S2 Table. Solo peptides of *Leishmania donovani* identified in urine of Indian and Sudanese patients with visceral leishmaniasis.

Sample	Experimental origin of peptide	Peptide	Parent protein	UniProtKB / GenBank accession
**India VL urine**	Dish 8 and 35	VTALEENIEAALR	Hypothetical protein, conserved	LdBPK_160420 / XP_003859730.1
	Dish 8	VTLLDR	3-oxoacyl-(acyl-carrier protein) reductase, putative	LdBPK_242110 / XP_003861301.1
	Dish 8 and 35	LSLEPR	Hypothetical protein, conserved	LdBPK_354400 / XP_003865018.1
	Dish 8	LVEEFHFSK	MP44, putative	LdBPK_270350 / XP_003861892.1
	Dish 8	ESPSPWVR	Hypothetical protein, conserved	LdBPK_281920 / XP_003862291.1
	Dish 8	RVAVVLEGR	Hypothetical protein, conserved	LdBPK_363030 / XP_003865418.1
	Dish 35	SELDARK	Hypothetical protein, conserved	LdBPK_100630 / XP_003858899.1
	Dish 35	ITQLVQLMK	Hypothetical protein, unknown function	LdBPK_312110 / XP_003863299.1
	Dish 35	CFNDDIQGTGAVIAAGFLNAVK	Malic enzyme	LdBPK_240780 / XP_003861168.1
	Dish 35	YDAASQIAILSMER	Enoyl-CoA hydratase/isomerase family protein, conserved	LdBPK_350360 / XP_003864629.1
**Sudan VL urine**	VLu	AGNVSINQHEGQR	RNA binding protein, putative	LdBPK_190290 / XP_003860208.1
	VLu/uAg*	MEEYLHSKDSAEQR	Hypothetical protein, conserved	LdBPK_220520 / XP_003860802.1
	VLu/uAg*	NSSSRAKADYKPSSSR	Hypothetical protein, conserved	LdBPK_333140 / XP_003864138.1
	uAg	SPASASVEAVGAAAFAR ****	Hypothetical protein, conserved	LdBPK_091050 / XP_003858784.1

	VLu	VWTKDLSQMK	Hypothetical protein, conserved	LdBPK_353990 / XP_003864977.1
	uAg	IAASVPSLR	Coatomer gamma subunit, putative	LdBPK_282820 / XP_003862380.1
	VLu/uAg*	NHAKQLYMR	Protein kinase, putative	LdBPK_210190 / XP_003860536.1
	uAg	GTYEVICR ****	Hypothetical protein, conserved	LdBPK_151420 / XP_003859665.1
	uAg	QAETALVNR ****	Hypothetical protein, conserved	LdBPK_303110 / XP_003863022.1

**** These peptides were assigned to *L. donovani* proteins from mass spectrometry data after a Mascot search of an *L. donovani*-only database. All other peptides were assigned to *Leishmania* proteins after a simultaneous *L. donovani* and human database search. However, these indicated peptides had very high homology to *Leishmania* proteins in subsequent homology searches and were therefore retained.

*Some gel fragments analysed by mass spectrometry spanned two sample types.
