# Supplementary material for: Isolation and characterisation of Leishmania donovani protein antigens from urine of visceral leishmaniasis patients
Source: PLoS One. 2020 Sep 14;15(9):e0238840. doi: 10.1371/journal.pone.0238840 (PMC7489519; doi:10.1371/journal.pone.0238840)
Supplement: S3 Table — (PDF) [file pone.0238840.s005.pdf]

S3 Table. Number of epitope peptides of ≥8 amino acids in *Leishmania donovani* proteins identified from VL patients' urine.

**VL urine origin and method used**	**Protein name (UniProtKB accession)**	**Number of predicted epitopes (length range in amino acids) at two epitope cut-off scores**		**Number of N-linked glycosylation sites**
		**0.65**	**0.55**	
India (immunopanning)	Hypothetical protein (LdBPK_191140)	6 (8-10 aa)	48 (8-86 aa)	1
	40S ribosomal protein S9 (LdBPK_070760)	4 (8-17 aa)	25 (8-142 aa)	0
	Hypothetical protein (LdBPK_323250)	8 (8-24 aa)	18 (8-64 aa)	1
	Protein kinase (LdBPK_262110)	12 (8-14 aa)	23 (9-284 aa)	3
Sudan (immunocapture and western blot)	Hypothetical protein ‡ (LdBPK_160110)	0	1 (29 aa)	0
	Protein kinase (LdBPK_351070)	3 (9-25 aa)	4 (15-149 aa)	1
Total		33	119	4 proteins

‡ The lower epitope score threshold was used for this protein due to absence of epitopes >8 aa at the higher score threshold.
